# ﻿The first complete mitochondrial genome of a *Lethrus* species (Coleoptera, Geotrupidae) with phylogenetic implications

**DOI:** 10.3897/zookeys.1236.138465

**Published:** 2025-04-24

**Authors:** Réka Zsófia Bubán, Renáta Bőkényné Tóth, Csongor Freytag, Gábor Sramkó, Zoltán Barta, Nikoletta Andrea Nagy

**Affiliations:** 1 One Health Institute, University of Debrecen, Nagyerdei krt. 98, H-4032 Debrecen, Hungary University of Debrecen Debrecen Hungary; 2 Department of Botany, University of Debrecen, Egyetem tér 1, H-4032 Debrecen, Hungary University of Debrecen Debrecen Hungary; 3 HUN-REN-UD Conservation Biology Research Group, University of Debrecen, Egyetem tér 1, H-4032 Debrecen, Hungary University of Debrecen Debrecen Hungary; 4 Department of Evolutionary Zoology and Human Biology, University of Debrecen, Egyetem tér 1, H-4032 Debrecen, Hungary University of Debrecen Debrecen Hungary; 5 HUN-REN-UD Behavioural Ecology Research Group, Department of Evolutionary Zoology, University of Debrecen, Egyetem tér 1, H-4032 Debrecen, Hungary University of Debrecen Debrecen Hungary

**Keywords:** Circular mitogenome, phylogenetic relationship, phytophagous, Scarabaeoidea, third-generation sequencing

## Abstract

The flightless beetle genus *Lethrus* Scopoli, 1777 (Geotrupidae, Scarabaeoidea) has a large distribution area throughout Eurasia and is characterized by many species, especially in Middle Asia. Despite this diversity and the potential importance as models for speciation, *Lethrus* species are underrepresented in molecular databases. To fill this gap, we report the complete mitochondrial genome of *Lethrusscoparius* obtained using third-generation sequencing technology. The circular mitogenome is 24,944 bp long and has a structure characteristic of coleopterans. It contains 37 genes, including 13 protein-coding genes, two ribosomal RNA genes, 22 transfer RNA genes, and an A+T-rich non-coding region, the control region between the 12S rRNA and tRNA-*Ile* (GAU). The phylogenetic analysis of the superfamily Scarabaeoidea placed *L.scoparius* in the monophyletic family Geotrupidae, which is related to the family Scarabaeidae. The assembled mitochondrial genome is a valuable new genomic resource in the genus *Lethrus* and contributes to a better understanding of the evolutionary history of the genus and the entire family Geotrupidae.

## ﻿Introduction

The family Geotrupidae is a relatively small, monophyletic group within the large and diverse superfamily Scarabaeoidea (Coleoptera) (Vuts et al. 2014). The species belonging to this group are divided into three subfamilies: Geotrupinae, Taurocerastinae, and Lethrinae (Cunha et al. 2011). The latter group consists of only one genus, *Lethrus* Scopoli, 1777, which comprises about 130 species and is subdivided into several subgenera (Shapovalov 2022). These beetles are flightless and have fused elytra. Most species exhibit sexual dimorphism, in which the males have a pair of tusk-like appendages on the ventral side of their mandibles that the females do not bear (Bagaturov and Nikolajev 2015). Another distinctive feature of these species compared to their geotrupid relatives is that they are phytophagous, feeding on fresh plant material of various species (Shapovalov 2022).

*Lethrus* is one of the most species-rich genera within the family Geotrupidae, with a wide distribution range in the Palearctic, especially in semi-arid habitats from Central and Southeastern Europe to China and Mongolia (Král et al. 2013; Bagaturov and Nikolajev 2015; Shapovalov 2022). Although some species occur sympatrically, most *Lethrus* species have small allopatric distributions (e.g. Král et al. 2013; Shapovalov 2022). One possible explanation for this patchy distribution pattern is the flightless nature of these species that might have limited their dispersal ability and thus triggered an increased speciation rate within the genus, similar to other examples (Ikeda et al. 2012; Salces-Castellano et al. 2021), especially in the regions of Middle Asia and the Balkans (Scholtz 2000; Bagaturov and Nikolajev 2015; Tóth et al. 2019). Despite the relatively high number of species in the genus and the unique life history traits, there are few molecular resources available for this taxon, both for phylogenetic and ecological genetic purposes.

In a study aimed at elucidating the phylogenetic relationships between the European *Lethrus* species based on targeted mitochondrial and nuclear loci, molecular data on Middle Asian species included as outgroup taxa were presented for the first time (Tóth et al. 2019). They were placed at the basal position of the genus. In this study, one species with a limited distribution area, *Lethrusscoparius* Fischer von Waldheim, 1822, was found to represent a clade subordinate to the European species. This species lives in the western Tian Shan, where individuals inhabit the plains and foothills (Nikolajev 2003). Based on the morphology of the adults, *L.scoparius* shows remarkable differences from the other species of the genus and was therefore described as the only member of the subgenus Autolethrus and proposed for further molecular studies to clarify its relationship with other *Lethrus* species (Bagaturov and Nikolajev 2015).

Mitochondrial genomes are important resources for phylogenetic, phylogeographic and population genetic studies at broad taxonomic scales (Cameron 2014; Kern et al. 2020; Zhao et al. 2021) because they are relatively small, simple in structure, have conserved gene arrangements, stable compositions and are maternally inherited (Kern et al. 2020; Jia et al. 2023). Although complete, circular mitochondrial sequences are available for many Scarabaeoidea species, they are scarce in the family Geotrupidae. The sole mitochondrial genome from the genus is that of *Lethrusapterus* Laxmann, 1770 (Nagy et al. 2021), which is fully annotated but incomplete. *De novo* assemblies of mitogenomes using short reads in many cases resulted in partial genomes, without an assembled A+T-rich non-coding region, also known as the control region (CR), and even without genes located near this highly repetitive sequence (e.g. Song and Zhang 2018). With the advances of long-read sequencing platforms, the incompleteness of such assemblies can be addressed.

In this study, we used Oxford Nanopore Technologies (ONT) long-read sequencing to determine the complete mitochondrial genome of *Lethrusscoparius*. It is the first complete mitogenome of the genus and one of the two available circularly assembled mitogenomes in the family Geotrupidae. We describe the structure of the genome, codon usage, nucleotide composition, and gene order. Additionally, we compare these features with the other complete mitochondrial genomes within the superfamily and examine the phylogenetic relationships of the taxa. The mitochondrial genome presented provides valuable information for future work to uncover the internal relationships within the genus *Lethrus* and at higher taxonomic levels.

## ﻿Methods

### ﻿Sample collection and DNA extraction

An adult male of *Lethrusscoparius* was collected on 1 May 2023 in Tian Shan, Kazakhstan (42°23.96'N, 70°27.66'E) and housed at the Department of Evolutionary Zoology and Human Biology, University of Debrecen, Hungary. The species was identified by morphological characteristics (Bagaturov and Nikolajev 2015) and stored in 96% ethanol at 4 °C until DNA extraction. High-molecular-weight (HMW) DNA was isolated from the whole body using a conventional DNA extraction method. Briefly, a lysis buffer with a final concentration of 3 mM CaCl2, 2% sodium dodecyl sulfate (SDS), 40 mM dithiothreitol (DTT), 250 μg/ml proteinase K, 100 mM Tris buffer (pH = 8.0) and 100 mM NaCl was used (Gilbert et al. 2007). Cell lysis lasted 2 h at 55 °C, during which the sample was inverted several times. After incubation, the sample was centrifuged at 14,000 *g* for 1 min and the supernatant was transferred to a clean 2 ml tube to avoid contamination with chitin in subsequent steps. Then the sample was incubated with 15 μl (10 mg/ml) RNase A (Roche, Switzerland) for 10 minutes at 37 °C. In the next step, 0.5 volume of 7.5 M ammonium acetate was added, and the sample was incubated at +4 °C for 10 min. After incubation, 0.5 volume of a chloroform-isoamyl alcohol mixture (24:1) was added and the sample was incubated for 10 min at room temperature. The sample was centrifuged at 10,000 *g* for 3 min, then the supernatant was transferred to a clean 2 ml tube. After this step, 1 volume of room temperature isopropanol was added, and the sample was incubated at +4 °C for 15 min. After the sample was centrifuged at 10,000 *g* for 3 min, the supernatant was carefully removed from the pellet. The pellet was washed twice with 1 volume of room temperature 70% ethanol and centrifuged at 10,000 *g* for 3 min in between. The pellet was air-dried at room temperature and then dissolved in 65 μl of 10 mM Tris-HCl (pH = 8.0).

The nucleic acid concentration and purity of the sample were measured using a NABI UV/Vis Nano Spectrophotometer (MicroDigital Co., Ltd, Korea). The integrity of DNA was checked by TBE agarose gel (1%) electrophoresis.

Residual RNA contamination was removed by an additional 30-min digestion step at 37 °C with RNase Cocktail™ Enzyme Mix (0.1 U RNase A and 4 U Rnase T1) (Invitrogen, USA). Further purification steps were performed with 0.6 volume of Ampure XP (Beckman Coulter, USA) according to the manufacturer’s instructions. To remove short DNA fragments, 0.64 volume of Long Fragment Buffer (Oxford Nanopore Technologies, UK) were also added. The decontaminated DNA was dissolved in 20 μl of nuclease-free water (Omega Bio-tek, Inc., USA). The concentration and purity of DNA were checked using a NanoDrop One (Thermo Fisher Scientific Inc., USA), a Qubit 4 Fluorometer (Thermo Fisher Scientific Inc., USA) with the 1x dsDNA High Sensitivity Kit (Thermo Fisher Scientific Inc., USA) and TBE agarose gel electrophoresis (1%).

### ﻿Library construction and sequencing

The third-generation sequencing library (ONT) was prepared from 1 μg of genomic DNA using the Ligation Sequencing Kit V14 (SQK-LSK114) (Oxford Nanopore Technologies, UK) according to the manufacturer’s recommendations. For DNA end repair, the NEBNext® Companion Module for Oxford Nanopore Technologies® Ligation Sequencing (NEB, E7180S) (New England Biolabs. Inc.) was used. 52 ng of the final library was loaded two times onto an R10.4 flow cell (FLO-MIN114). The sequencing run was performed using a MinION Mk1C device for 72 h.

### ﻿Genome assembly and annotation

The sequencing yielded 9.26 giga base pairs (Gbp) of raw data. The Super high-accuracy model with Guppy 6.5.7 (Wick et al. 2019) was used for basecalling. Raw sequencing reads have been deposited to the Sequence Read Archive (SRA) under the accession number SRR28464392. The quality of the long reads was checked with the R script of MinIONQC 1.4.2 (Lanfear et al. 2019). Reads of the used DNA control strand (DNA CS) were removed with NanoLyse 1.2.0, then NanoFilt 2.8.0 (De Coster et al. 2018) was used to trim both ends of the reads (--headcrop 50, --tailcrop 50) and eliminate reads with low quality (mean quality cut-off 8 (-q 8)) or insufficient length (shorter than 500 bp (-l 500)). The cleaned and filtered reads were analyzed with NanoPlot 1.40.0 (De Coster et al. 2018). The quality filtering resulted in 2,751,077 clean reads with a total length of 6.79 Gbp and a read N50 of 4,124 bp.

Minimap 2.17 (Li 2018) was used to map the filtered reads to the mitochondrial genome of *Lethrusapterus* (Nagy et al. 2021), the closest relative with a fully annotated but incomplete mitochondrial genome. Reads covering more than 70% of the reference mitogenome were accepted as mitochondrial sequences. *De novo* assembly of the mitochondrial reads was performed using Flye 2.9 (Kolmogorov et al. 2019) with the predicted genome size set to 16 kbp and the assembly coverage set to 100 (-g 16k --asm-coverage 100). The assembled sequence was polished using Racon 1.4.10 (Vaser et al. 2017) and medaka 1.7.2 (https://github.com/nanoporetech/medaka) with the model r1041_e82_400bps_sup_g615. The annotation of the assembled mitochondrial genome was performed with MITOS2 (Donath et al. 2019) based on the invertebrate genetic code. The annotated mitochondrial genome was visualized using Proksee (Grant et al. 2023).

The AT-, and GC-skew of the sequences were calculated with the following formulas: AT-skew = (A – T) / (A + T); and GC-skew = (G – C) / (G + C) (Perna and Kocher 1995). Amino acid abundances and relative synonymous codon usage (RSCU) values of mitochondrial protein-coding genes (PCGs) were analyzed with Ezcodon (Lee 2018) implemented in EZmito Server (Cucini et al. 2021) using the invertebrate mitochondrial genetic code.

### ﻿Phylogenetic analysis

The complete circular and annotated mitochondrial genomes of the superfamily Scarabaeoidea were downloaded from NCBI GenBank on 13 January 2025 (Suppl. material 1: table S1). Species represented the families Lucanidae (three subfamilies), Geotrupidae, Passalidae, Scarabaeidae (six subfamilies), and Trogidae. The mitogenome of the closest relative, *Lethrusapterus*, was also included, as it contains all coding regions needed to reconstruct the phylogenetic tree. In addition, the mitochondrial genomes of *Apateticaglabra* (Staphylinoidea), *Diamesusosculans* (Staphylinoidea), and *Sphaeridiumbipustulatum* (Hydrophiloidea) were used as outgroup samples. The nucleotide sequences of PCGs and ribosomal RNA (rRNA) coding genes were collected from the annotations. The sequences of PCGs were aligned with MACSE 2.07 (Ranwez et al. 2018) using the invertebrate mitochondrial code (-gc_def 5), and MUSCLE 3.8.1551 (Edgar 2004) was used to align the rRNA gene sequences. The aligned nucleotide sequences were merged using AMAS.py (Borowiec 2016) to obtain a 13,812 bp long alignment. Phylogenetic reconstruction was performed using IQ-TREE 2.0.3 (Nguyen et al. 2015). All genetic regions were defined as distinct partitions and ModelFinder Plus (Kalyaanamoorthy et al. 2017) was used to find the best fitting partition scheme for the dataset. Branch support was assessed using the SH-like approximate likelihood ratio test (SH-aLRT) (Guindon et al. 2010) and the ultrafast bootstrap (UFBoot) (Minh et al. 2013) with 1000 replicates.

## ﻿Results

### ﻿General characteristics of the mitogenome

**Figure 1.**
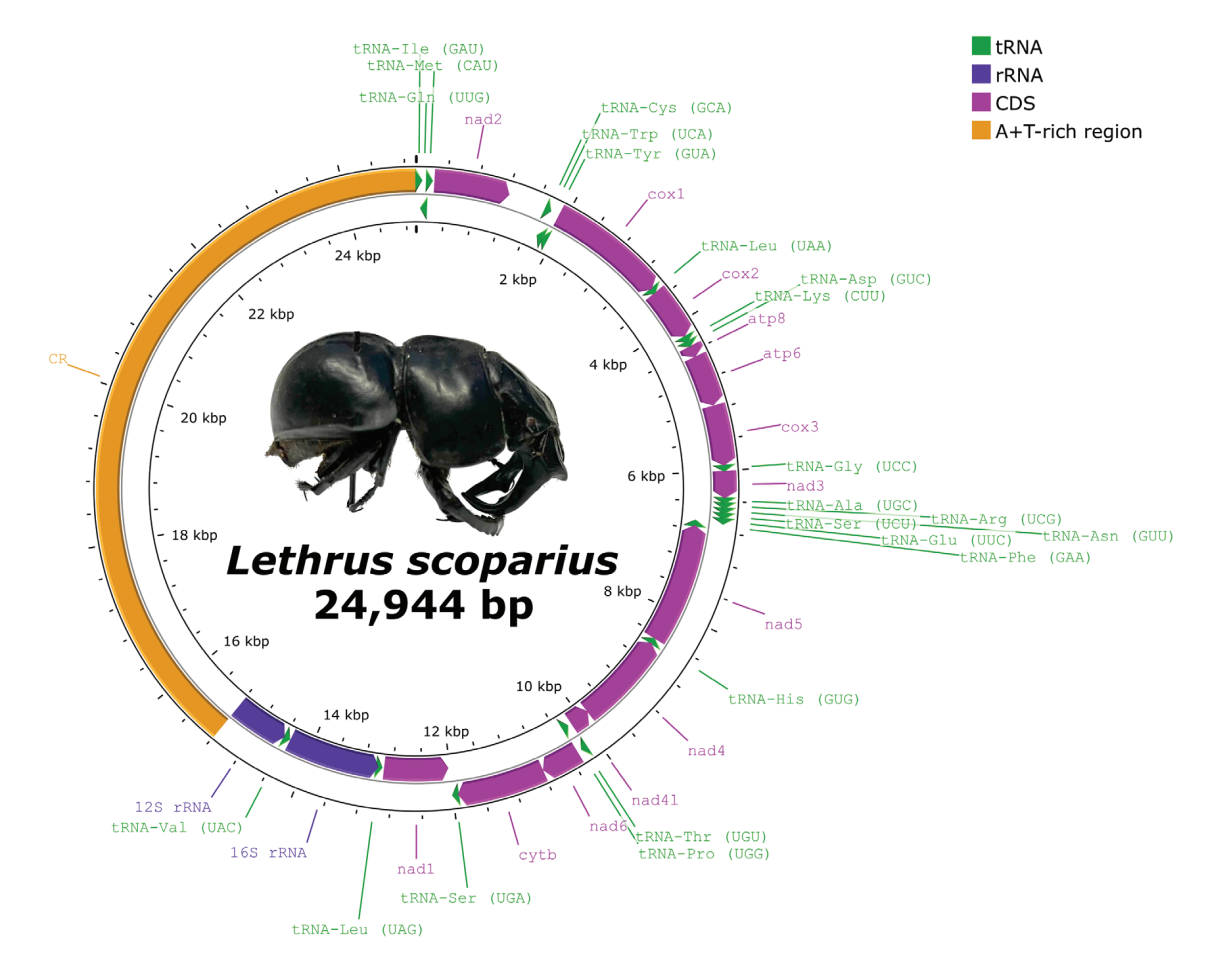
The circular mitochondrial genome map of *Lethrusscoparius*. Genes on the outer circle are located on the heavy strand, whereas the inner circle represents genes on the light strand. Protein-coding genes are shown in purple, tRNA genes in green, rRNA genes in blue, and the control region is represented in orange. Photo taken by RZB.

The assembled mitochondrial genome of *Lethrusscoparius* was complete, circular, and 24,944 bp long. The mitogenome contained 13 protein-coding genes (PCG), two ribosomal RNA genes (rRNA), 22 transfer RNA genes (tRNA), and the control region (CR) (Fig. 1, Table 1). Most of the PCGs and tRNA genes as well as the CR were encoded on the heavy strand, whereas the remaining four PCGs (*nad1*, *nad4*, *nad4l* and *nad5*), eight tRNA genes (tRNA-*Cys* (GCA), tRNA-*Gln* (UUG), tRNA-*His* (GUG), tRNA-*Leu* (UAG), tRNA-*Phe* (GAA), tRNA-*Pro* (UGG), tRNA-*Tyr* (GUA), tRNA-*Val* (UAC)), and the two rRNA genes were located on the light strand (Table 1).

**Table 1.** Annotation of the mitochondrial genome of *Lethrusscoparius*. The Strand column shows the orientation of the genes, where the + marks the heavy strand and – denotes the light strand. The ovl/nc column indicates the overlapping or intergenic (non-coding) nucleotides, where the positive value means intergenic nucleotides and the negative value indicates overlapping nucleotides.

Gene	Region (bp)	Strand	Length (bp)	ovl/nc	Codons	
					Start	Stop
tRNA-*Ile* (GAU)	1–67	+	67	0		
tRNA-*Gln* (UUG)	68–136	–	69	−1		
tRNA-*Met* (CAU)	136–204	+	69	30		
*nad2*	235–1,218	+	984	499	ATT	TAA
tRNA-*Trp* (UCA)	1,718–1,784	+	67	−8		
tRNA-*Cys* (GCA)	1,777–1,839	–	63	1		
tRNA-*Tyr* (GUA)	1,841–1,908	–	68	−2		
*cox1*	1,907–3,445	+	1,539	−5	TCG	TAA
tRNA-*Leu* (UAA)	3,441–3,504	+	64	−1		
*cox2*	3,504–4,187	+	684	3	ATG	TAA
tRNA-*Lys* (CUU)	4,191–4,261	+	71	0		
tRNA-*Asp* (GUC)	4,262–4,328	+	67	0		
*atp8*	4,329–4,484	+	156	−7	ATC	TAA
*atp6*	4,478–5,149	+	672	1	ATG	TAA
*cox3*	5,151–5,938	+	788	−1	ATG	TA(A)
tRNA-*Gly* (UCC)	5,938–6,003	+	66	0		
*nad3*	6,004–6,357	+	354	−2	ATA	TAG
tRNA-*Ala* (UGC)	6,356–6,421	+	66	−1		
tRNA-*Arg* (UCG)	6,421–6,486	+	66	3		
tRNA-*Asn* (GUU)	6,490–6,554	+	65	0		
tRNA-*Ser* (UCU)	6,555–6,621	+	67	0		
tRNA-*Glu* (UUC)	6,622–6,688	+	67	−2		
tRNA-*Phe* (GAA)	6,687–6,752	–	66	−3		
*nad5*	6,750–8,463	–	1,714	9	ATT	T(AA)
tRNA-*His* (GUG)	8,473–8,538	–	66	−1		
*nad4*	8,538–9,874	–	1,337	−7	ATG	TA(A)
*nad4l*	9,868–10,158	–	291	2	ATG	TAA
tRNA-*Thr* (UGU)	10,161–10,224	+	64	0		
tRNA-*Pro* (UGG)	10,225–10,288	–	64	2		
*nad6*	10,291–10,794	+	504	−1	ATT	TAA
*cytb*	10,794–11,936	+	1,143	−2	ATG	TAG
tRNA-*Ser* (UGA)	11,935–12,000	+	66	17		
*nad1*	12,018–12,929	–	912	40	ATA	TAA
tRNA-*Leu* (UAG)	12,970–13,030	–	61	−23		
16S rRNA	13,008–14,320	–	1,313	18		
tRNA-*Val* (UAC)	14,339–14,408	–	70	0		
12S rRNA	14,409–15,192	–	784	0		
CR	15,193–24,944	+	9,752	—		

The overall base composition of the mitogenome was 41.3% A, 13.6% C, 7.9% G, and 37.2% T, and the A+T content was 78.48%. The AT-skew was slightly positive: 0.052, and the GC-skew was negative: −0.268 (Suppl. material 1: table S2). Descriptive statistics of the protein-coding genes, transfer RNA genes and ribosomal RNA genes, including the amino acid frequency and the codon usage, length distribution and base content are detailed in the Suppl. material 1.

**Figure 2.**
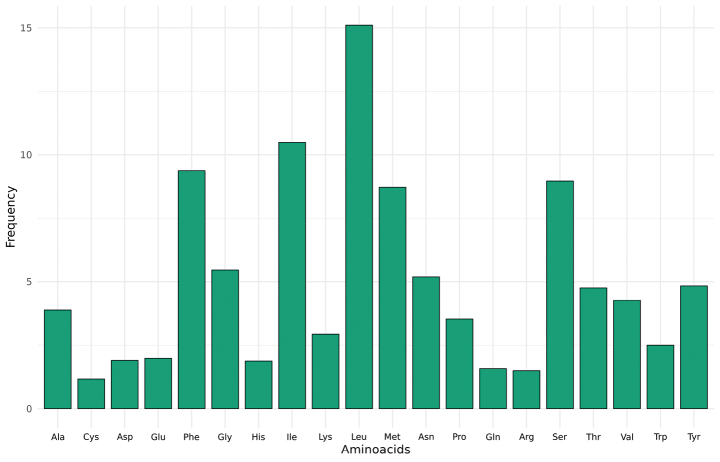
Amino acid frequency of the PCGs in the assembled mitochondrial genome of *Lethrusscoparius*. Three-letter amino acid code is shown on the X-axis and the frequency of amino acids in percentage on the Y-axis.

### ﻿Non-coding and overlapping regions

The largest non-coding region in the mitogenome of *L.scoparius* was the control region at 9,752 bp in length. The CR was located between the 12S rRNA and the tRNA-*Ile* (GAU) genes and had an A+T content of 80.2%. The base content of this region was 42.04% A, 13.33% C, 6.45% G, and 38.18% T. The AT-skew of the CR had a positive value of 0.048 and the GC-skew had a negative value of −0.348. The second largest non-coding region in the mitogenome had a length of 499 bp and was located between the nad2 and tRNA-*Trp* (UCA) genes. In addition, 12 other intergenic spacers were found that were between 1 bp and 40 bp in length (Table 1).

**Figure 3.**
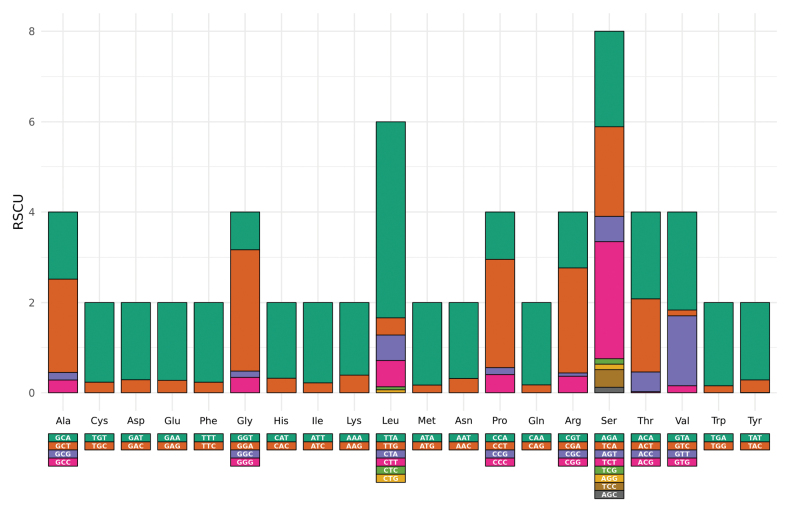
Relative synonymous codon usage (RSCU) of the PCGs in the assembled mitochondrial genome of *Lethrusscoparius*. Codon families and the three-letter amino acid codes are indicated on the X-axis and RSCU values on the Y-axis. Different colors of the bars represent different codons coding for the amino acid.

Several overlapping regions were observed in the mitogenome. The length of these sequences ranged from 1 bp to 23 bp (Table 1). The largest overlapping region was located between the tRNA-*Leu* (UAG) and the 16S rRNA genes. A 7-bp overlap with the “ATGATAA” motif was found between the PCGs*atp8* and *atp6*. In addition, an 8-bp overlap with the “AAGCCTTA” motif was detected between tRNA-*Trp* (UCA) and tRNA-*Cys* (GCA). Finally, an overlap with the “TTAACAT” motif was found between *nad4* and *nad4l*.

### ﻿Phylogenetic analysis

**Figure 4.**
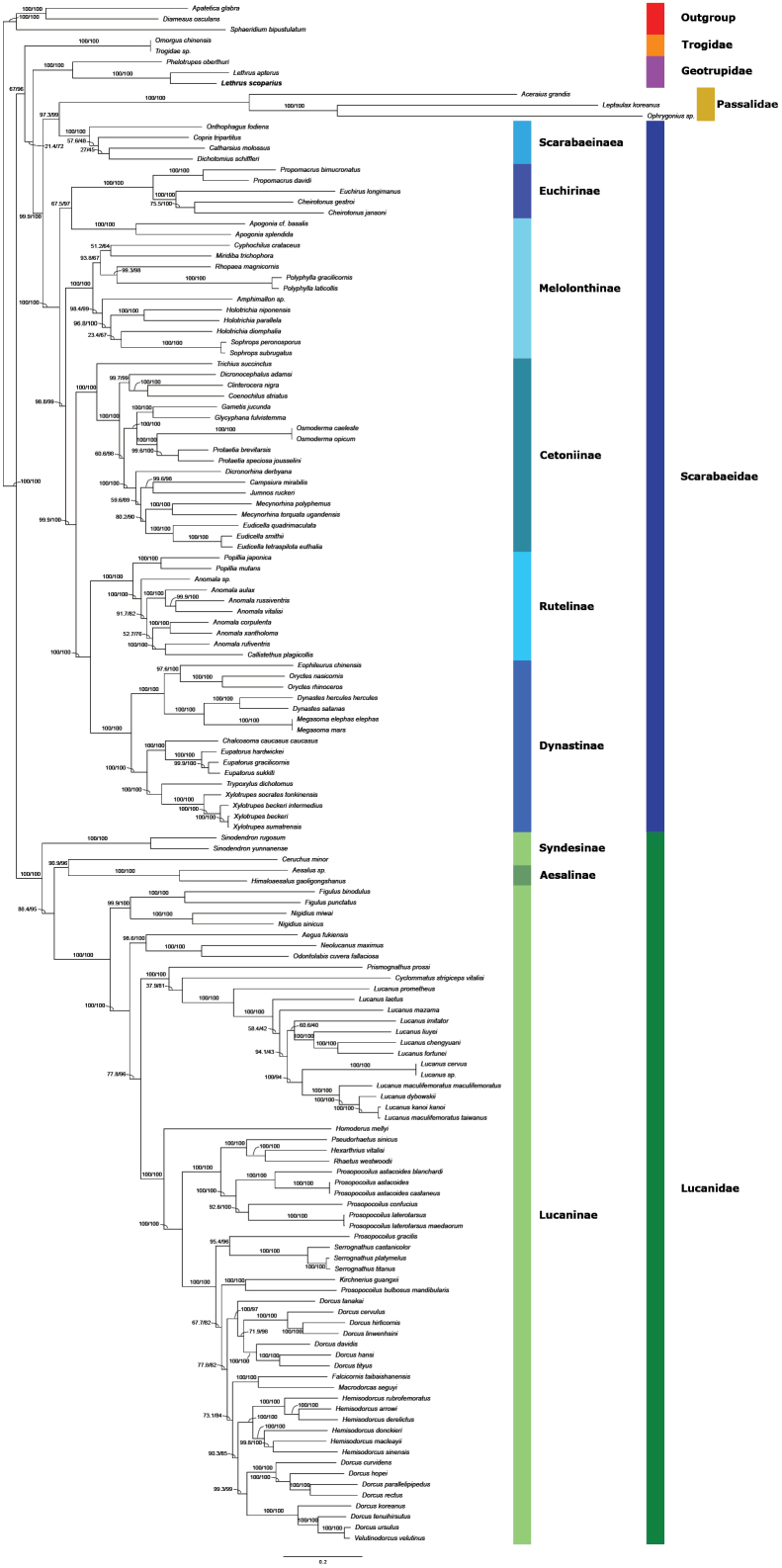
The reconstructed phylogenetic tree of the superfamily Scarabaeoidea based on species with a complete circular and annotated mitochondrial genome in the NCBI GenBank database (split into two parts). *Apateticaglabra*, *Diamesusosculans* and *Sphaeridiumbipustulatum* were used as an outgroup. This maximum-likelihood tree was based on the nucleotide sequences of 13 mitochondrial PCGs and two rRNA genes. Branch supporting was evaluated with SH-like approximate likelihood ratio test (SH-aLRT) and ultrafast bootstrap (UFBoot) with 1000 replicates. Colors represent the respective subfamilies (left panel) and families (right panel). *Lethrusscoparius* is highlighted in bold.

We performed a maximum-likelihood (ML) phylogenetic tree reconstruction based on the nucleotide sequences of the PCGs and rRNA genes of 145 species of the superfamily Scarabaeoidea (Fig. 4). Our analysis revealed two monophyletic groups in addition to the outgroup, one containing the representatives of the family Lucanidae and the other consisting of the species of all four other families. Within the Lucanidae clade, all three subfamilies formed separate monophyletic clusters with the following branching: (Syndesinae (Aesalinae + Lucaninae)). The structure of the other clade was (Trogidae (Geotruipdae (Scarabaeidae + Passalidae))). Within the family Scarabaeidae, the subfamilies Rutelinae and Dynastinae were found to be monophyletic sister groups with the closest relationship to the Cetoniinae. The species of the Melolonthinae were clustered into a paraphyletic group with two main branches. One of these was a sister group to the clade ((Rutelinae + Dynastinae) Cetoniinae), the other to the subfamily Euchirinae. Interestingly, the paraphyly of the family Scarabaeidae was a result of the cluster consisting of the subfamily Scarabaeinae and the family Passalidae. Within the family Geotrupidae, the two *Lethrus* species were found to have a sister relationship to the third representative of the family, *Phelotrupesoberthuri* Boucomont, 1905.

## ﻿Discussion

*Lethrusscoparius* is a rare Middle Asian earth-boring beetle from the genetically poorly characterized family Geotrupidae. Although *Lethrus* is one of the most species-rich genera in this family, molecular information on this scarabaeoid genus remains limited. In this study, we present the mitochondrial genome of *L.scoparius*, the first complete and well-characterized mitogenome of the genus. The mitogenome is 24,944 bp long and contains 37 genes (13 protein-coding genes, two ribosomal RNA genes and 22 transfer RNA genes) and a control region. The length of our assembled mitogenome is comparable with the elongated genomes found in Hercules beetles (*Dynastes*) (Morgan et al. 2022) and seed beetles (*Callosobruchus*) (Sayadi et al. 2017). The observed arrangement of genes was identical to the order of the insect ancestral mitochondrial genes (Sheffield et al. 2008). The nucleotide sequence of the genome showed a characteristically high A+T content, as well as a positive AT-skew and a negative GC-skew.

We found several intergenic spacers and overlapping regions of different lengths. Of the observed overlaps, three motifs were proved to be conserved between different arthropod groups. The overlapping motif “ATGATAA” between PCGs*atp8* and *atp6* was also observed in other coleopteran mitogenomes (Wang and Tang 2017, 2018; Shi et al. 2023). In addition, an “AAGCCTTA” overlap motif was found between tRNA-*Trp* (UCA) and tRNA-*Cys* (GCA), similar to several other insect mitochondrial genomes (Wang and Tang 2017; Wang et al. 2018; Cao et al. 2019). Finally, the overlap of the “TTAACAT” motif between *nad4* and *nad4l* is characteristic of several other arthropod mitogenomes (Huang et al. 2015; Wang et al. 2016, 2018).

To reconstruct the phylogenetic relationships within the superfamily, we used the sequences of all Scarabaeoidea species with a publicly available complete, circular, and fully annotated mitochondrial genome at the date of completing this work. The dataset comprised 145 species resulting in currently the most comprehensive phylogenetic analysis of the superfamily. The basic structure of our reconstructed phylogenetic tree was similar to the phylogenetic relationships found by Ahrens et al. (2014) and Dietz et al. (2023). Previous studies investigating the relationships of the superfamily Scarabaeoidea mostly aimed to clarify the phylogeny within the Scarabaeidae and used members of the other families as outgroups (e.g. Gunter et al. 2016) or included only a few species from them (e.g. Ahrens et al. 2014; Song and Zhang 2018; Dietz et al. 2023). Within the family Scarabaeidae, the subfamily Melolonthinae appeared to be paraphyletic due to the presence of Euchirinae as sister to one of the Melolonthinae lineages. This is in line with the results of Ahrens et al. (2014) and Dietz et al. (2023), who found similar evolutionary relationships based on nuclear and mitochondrial markers. This supports the concept that the species forming the subfamily Euchirinae should be placed in the tribe Euchirini within Melolonthinae (Song and Zhang 2018; Ayivi et al. 2021; Guo et al. 2022). Although a recent study that published the mitogenome of the two *Propomacrus* species concluded that the subfamily Euchirinae forms a separate monophyletic cluster (Yi et al. 2024), the *Apogonia* species that caused the paraphyly within the subfamily Melolonthinae were not included in their analysis. Nevertheless, this taxonomic question would warrant further investigation with an improved set of markers to clarify the relationships between these two taxa.

Another interesting paraphyletic structure was found in our phylogenetic tree between the passalids and the Scarabaeidae. Although this exact relationship has not yet been hypothesized by others, the uncertain position of the family Passalidae is a known problem (see e.g. Dietz et al. 2023). All three members of this family that we included in our analysis were placed on extremely long branches, suggesting long-branch attraction bias – a common phenomenon in mitochondrial sequences (Timmermans et al. 2016). Other recent studies (Gunter et al. 2016; Guo et al. 2022; Dietz et al. 2023) have also placed representatives of the family on long branches, which could be addressed by increasing the number of passalid species with complete mitochondrial sequences.

Our results indicate that Geotrupidae and the clade of Scarabaeidae + Passalidae are sister groups. In the most recent studies, the family Geotrupidae has been considered to be the sister group to Scarabaeidae (Ahrens et al. 2014), Bolboceratidae (Song and Zhang 2018; Dietz et al. 2023), and Trogidae plus Glaphyridae (Gunter et al. 2016). The results of Guo et al. (2022) show that the family Geotrupidae occupies different positions depending on the phylogenetic analysis method, although they only used this taxon as an outgroup in their study. Furthermore, the phylogenetic relationships within the family Geotrupidae have so far been analyzed mainly on the basis of larval morphology (Scholtz and Browne 1996; Verdú et al. 2004). Moreover, phylogenetic studies based on molecular markers have not yet included *Lethrus* species as representatives of the family Geotrupidae. Therefore, the mitochondrial sequence of *L.scoparius* is a useful contribution to corroborate the results found based on morphological characters.

The mitochondrial genome presented here is one of the few Scarabaeoidea mitogenomes generated using long-read sequencing technologies (Filipović et al. 2021; Morgan et al. 2022). The advantages of these methods could compensate for the shortcomings of the widely used short-read sequencing platforms. According to the results of Baeza et al. (2024), the latest Oxford Nanopore long-read sequencing chemical methods lead to a very low sequencing error rate, making the short-read polishing step in the assembly process redundant. With this method, the assembly of the non-coding control region, which often contains repetitive motifs, could be performed more accurately (Filipović et al. 2021). Elongated control regions reported in coleopteran species contain several tandem repeats, which have been proposed as informative markers for population genetic studies due to their higher evolutionary rate (Sayadi et al. 2017; Morgan et al. 2022). In addition, both Hercules beetles (Morgan et al. 2022) and seed beetles (Sayadi et al. 2017) have been shown to express specific sites of the control region using transcriptome sequencing data. Although the expression level was very low in both cases, these results suggest a role for these repeat sequences in a mitochondrial function. Our assembly is a novel example of the expansion of control regions in the Scarabaeoidea and suggests that it may be worthwhile to revisit the already assembled mitogenomes to detect expanded control regions and examine their tandem repeat content in the superfamily.

## ﻿Conclusions

Among the members of the family Geotrupidae, our assembly of the mitogenome of *Lethrusscoparius* and the mitogenome of *Phelotrupesoberthuri* are the only representatives with circularly assembled and fully annotated complete mitochondrial genomes. This sequence provides valuable molecular resources for understanding the phylogeny of this family and the molecular evolution of the mitochondrial genome within the superfamily Scarabaeoidea, and a good example of the high efficiency of third-generation sequencing techniques in assembling mitochondrial genomes.

## ﻿Additional information

### Author ORCIDs

Csongor Freytag https://orcid.org/0000-0002-3356-4182

Gábor Sramkó https://orcid.org/0000-0001-8588-6362

Zoltán Barta https://orcid.org/0000-0002-7121-9865

Nikoletta Andrea Nagy https://orcid.org/0000-0001-7771-830X

### Data availability

All of the data that support the findings of this study are available in the main text or Supplementary Information.

## Supplementary materials

Supplementary material 1 Supplementary data Data type pdf https://binary.pensoft.net/file/1314639This dataset is made available under the Open Database License (http://opendatacommons.org/licenses/odbl/1.0/). The Open Database License (ODbL) is a license agreement intended to allow users to freely share, modify, and use this Dataset while maintaining this same freedom for others, provided that the original source and author(s) are credited.Réka Zsófia Bubán, Renáta Bőkényné Tóth, Csongor Freytag, Gábor Sramkó, Zoltán Barta, Nikoletta Andrea Nagy
