# Supplementary material for: ﻿The first complete mitochondrial genome of a Lethrus species (Coleoptera, Geotrupidae) with phylogenetic implications
Source: Zookeys. 2025 Apr 24;1236:1–17. doi: 10.3897/zookeys.1236.138465 (PMC12046339; doi:10.3897/zookeys.1236.138465)
Supplement: Supplementary material 1 — Supplementary data [file zookeys-1236-001_article-138465__-s001.pdf]

Supplementary material

Sequence and phylogenetic implications of the mitochondrion of *Lethrus scoparius* (Coleoptera, Geotrupidae), the first complete mitochondrial sequence of the genus

Réka Zsófia Bubán[1,2], Renáta Bőkényné Tóth[1], Csongor Freytag[1], Gábor Sramkó[2,3], Zoltán Barta[4,5], Nikoletta Andrea Nagy[4,5]

[1]One Health Institute, University of Debrecen, Nagyerdei krt. 98, H-4032 Debrecen, Hungary
[2]Department of Botany, University of Debrecen, Egyetem tér 1, H-4032 Debrecen, Hungary
[3]HUN-REN-UD Conservation Biology Research Group, University of Debrecen, Egyetem tér 1, H-4032 Debrecen, Hungary
[4]Department of Evolutionary Zoology and Human Biology, University of Debrecen, Egyetem tér 1, H-4032 Debrecen, Hungary
[5]HUN-REN-UD Behavioural Ecology Research Group, Department of Evolutionary Zoology, University of Debrecen, Egyetem tér 1, H-4032 Debrecen, Hungary

Corresponding author: Nikoletta Andrea Nagy (nagy.nikoletta@science.unideb.hu)

Descriptive statistics of the genes in the mitochondrial genome of *Lethrus scoparius*

Protein-coding genes

The total length of the 13 PCGs was 11,087 bp, which corresponds to 44.41% of the total genome size. The average base content in the PCGs was 33.95% A, 12.30% C, 11.06% G, and 42.69% T. The average A+T content of the PCGs was 76.64% (ranging from 69.79% to 82.69%; Table S2), indicating that A and T were more abundant in all these genes than C and G. In addition, the negative value (-0.11) of the average AT-skew (ranging from -0.28 to 0.02) indicated that T was more abundant than A in the PCGs. The average GC-skewness of the PCGs was -0.05 (ranging from -0.56 to 0.39), i.e. C was more frequent than G, except for the nad1, nad4, nad4l and nad5 genes, where positive GC-skew values were observed (Table S2).

All PCGs started with an ATN codon, except cox1, which started with a TCG codon (Table 1). Eight of the 13 PCGs had the typical stop codon TAA, two (cytb and nad3) ended with TAG and three had abbreviated termination codons TA (cox3 and nad4) and T (nad5) (Table 1). Analysis of amino acid abundance (Fig. 2) and relative synonymous codon usage (RSCU) (Fig. 3) showed that the three most frequently encoded amino acids were leucine (Leu), isoleucine (Ile) and phenylalanine (Phe), while the least abundant amino acid was cysteine (Cys). Serine (Ser) and leucine (Leu) showed the greatest diversity with eight and six codons, respectively. In addition, the most abundant codons of the PCGs were UUA (leucine), GGA (glycine) and CCU (proline).

Transfer and ribosomal RNA genes

The length of the 22 tRNA genes ranged from 61 bp to 71 bp, and the total length of the tRNAs was 1,459 bp. The average base composition of the tRNA genes was 40.49% A, 8.68% C, 11.13% G, and 39.69% T. The average value of AT- and GC-skewness had slightly positive values: 0.009 (ranging from -0.15 to 0.22) and 0.128 (ranging from -0.50 to 0.64), respectively (Table S2). Prediction of the secondary structures of the tRNA genes (Fig. S1) revealed that all genes had a cloverleaf structure, except for the tRNA-Ser (UCU), where the dihydrouridine (DHU) arm was truncated, which has been frequently observed in insects (Cameron 2014; Zhao et al. 2020; Chen et al. 2022).

The length of the 12S rRNA gene was 784 bp, whereas the 16S rRNA gene was 1,313 bp long. The average nucleotide content of the two rRNA genes was 37.85% A, 6.39% C, 13.49% G and 42.27% T. The average AT-skew had a slightly negative value of -0.055 and the average GC-skew was positive: 0.358 (Table S2).

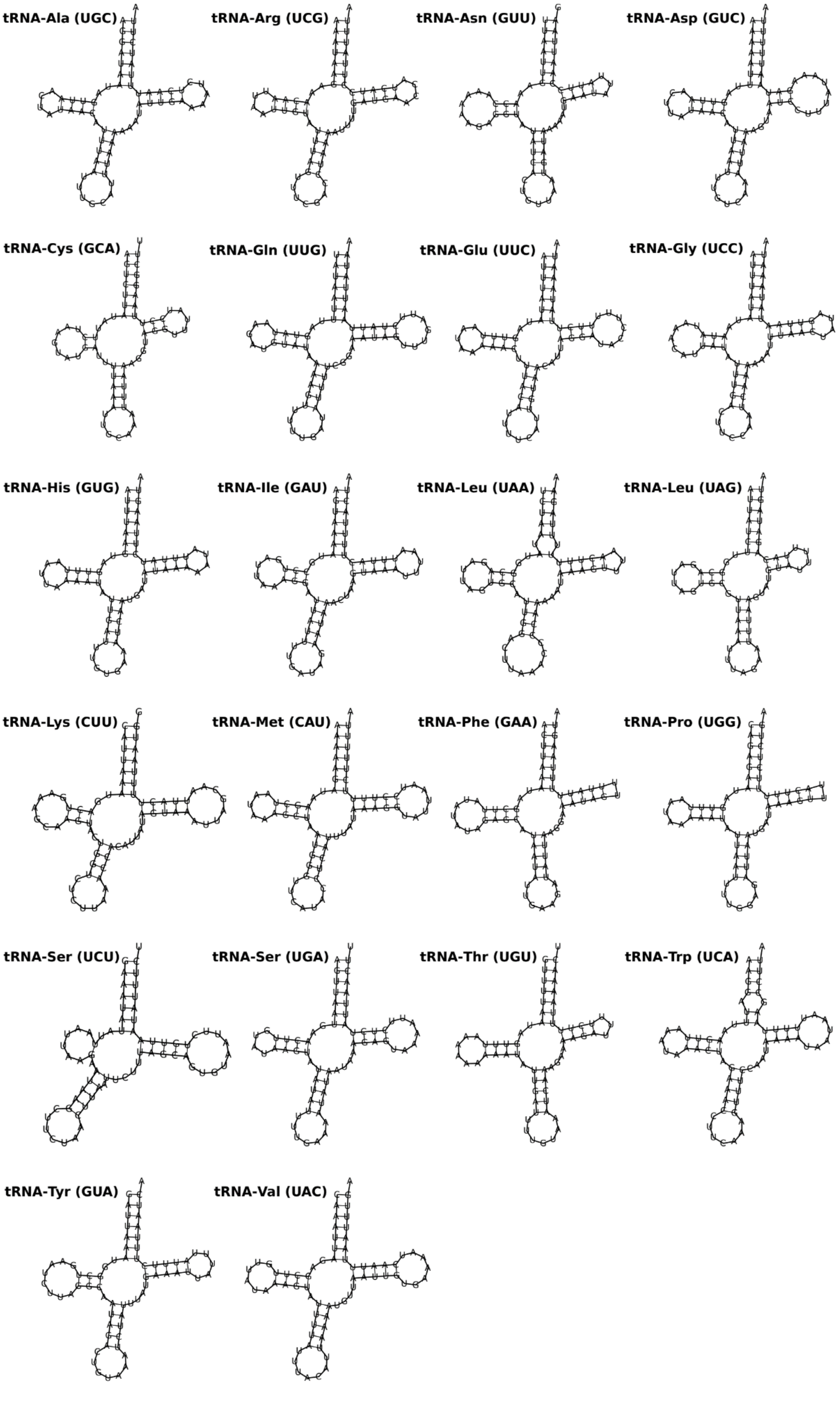

Figure S1. The predicted secondary structures of the tRNA genes in the assembled mitochondrial genome of *Lethrus scoparius*.

Table S1. List of the species and the accession numbers of their mitochondrial genomes used for the phylogenetic reconstruction.

Family	Subfamily	Species	GenBank accession number	Reference publication
Geotrupidae	-	*Lethrus apterus*	BK067253	This study
Geotrupidae	-	*Lethrus scoparius*	BK068120	Direct submission
Geotrupidae	-	*Phelotrupes oberthuri*	MT548773.1	Direct submission
Hydrophilidae	Sphaeridiinae	*Sphaeridium bipustulatum*	NC_028612.1	Direct submission
Lucanidae	Aesalinae	*Aesalus sp.*	MH120282.1	Direct submission
Lucanidae	Aesalinae	*Himaloaesalus gaoligongshanus*	NC_085546.1	Direct submission
Lucanidae	Lucaninae	*Aegus fukiensis*	NC_085524.1	Direct submission
Lucanidae	Lucaninae	*Cyclommatus strigiceps vitalisi*	NC_045068.1	Direct submission
Lucanidae	Lucaninae	*Dorcus cervulus*	NC_082960.1	Jafir et al. 2024
Lucanidae	Lucaninae	*Dorcus curvidens*	NC_082946.1	Jafir et al. 2024
Lucanidae	Lucaninae	*Dorcus davidis*	NC_082947.1	Jafir et al. 2024
Lucanidae	Lucaninae	*Dorcus hansi*	NC_043928.1	Direct submission
Lucanidae	Lucaninae	*Dorcus hirticornis*	NC_082962.1	Jafir et al. 2024
Lucanidae	Lucaninae	*Dorcus hopei*	NC_082948.1	Jafir et al. 2024
Lucanidae	Lucaninae	*Dorcus koreanus*	NC_054278.1	Kim et al. 2020
Lucanidae	Lucaninae	*Dorcus linwenhsini*	NC_082949.1	Jafir et al. 2024
Lucanidae	Lucaninae	*Dorcus parallelipipedus*	KT876887.1	Linard et al. 2016
Lucanidae	Lucaninae	*Dorcus rectus*	NC_082950.1	Jafir et al. 2024
Lucanidae	Lucaninae	*Dorcus tanakai*	NC_082951.1	Jafir et al. 2024
Lucanidae	Lucaninae	*Dorcus tenuihirsutus*	NC_045124.1	Chen et al. 2021
Lucanidae	Lucaninae	*Dorcus tityus*	NC_082952.1	Jafir et al. 2024
Lucanidae	Lucaninae	*Dorcus ursulus*	NC_045123.1	Chen et al. 2021
Lucanidae	Lucaninae	*Falcicornis taibaishanensis*	NC_082953.1	Jafir et al. 2024
Lucanidae	Lucaninae	*Figulus binodulus*	NC_045102.1	Lee et al. 2020
Lucanidae	Lucaninae	*Figulus punctatus*	NC_081970.1	Choi et al. 2024
Lucanidae	Lucaninae	*Hemisodorcus arrowi*	NC_082954.1	Jafir et al. 2024
Lucanidae	Lucaninae	*Hemisodorcus derelictus*	NC_082955.1	Jafir et al. 2024

Lucanidae	Lucaninae	*Hemisodorcus donckieri*	NC_082956.1	Jafir et al. 2024
Lucanidae	Lucaninae	*Hemisodorcus macleayii*	NC_082957.1	Jafir et al. 2024
Lucanidae	Lucaninae	*Hemisodorcus rubrofemoratus*	NC_082958.1	Jafir et al. 2024
Lucanidae	Lucaninae	*Hemisodorcus sinensis*	NC_082959.1	Jafir et al. 2024
Lucanidae	Lucaninae	*Hexarthrius vitalisi*	PP112342.1	Direct submission
Lucanidae	Lucaninae	*Homoderus mellyi*	PP085183.1	Jin et al. 2024
Lucanidae	Lucaninae	*Kirchnerius guangxii*	NC_048957.1	Zhai et al. 2020
Lucanidae	Lucaninae	*Lucanus cervus*	NC_060601.1	Direct submission
Lucanidae	Lucaninae	*Lucanus chengyuani*	MK878514.1	Wang et al. 2019
Lucanidae	Lucaninae	*Lucanus dybowskii*	PP140409.1	Direct submission
Lucanidae	Lucaninae	*Lucanus fortunei*	NC_044961.1	Cao et al. 2019
Lucanidae	Lucaninae	*Lucanus imitator*	PP140407.1	Direct submission
Lucanidae	Lucaninae	*Lucanus kanoi kanoi*	MK238348.1	Lin et al. 2017
Lucanidae	Lucaninae	*Lucanus laetus*	PP140408.1	Direct submission
Lucanidae	Lucaninae	*Lucanus liuyei*	PP110548.1	Direct submission
Lucanidae	Lucaninae	*Lucanus maculifemoratus maculifemoratus*	PP103655.1	Direct submission
Lucanidae	Lucaninae	*Lucanus maculifemoratus taiwanus*	MK250907.1	Direct submission
Lucanidae	Lucaninae	*Lucanus mazama*	NC_013578.1	Sheffield et al. 2009
Lucanidae	Lucaninae	*Lucanus prometheus*	NC_060603.1	Direct submission
Lucanidae	Lucaninae	*Lucanus sp.*	KT876903.1	Linard et al. 2016
Lucanidae	Lucaninae	*Macrodorcas seguyi*	NC_038212.1	Chen et al. 2018
Lucanidae	Lucaninae	*Neolucanus maximus*	NC_039652.1	Direct submission
Lucanidae	Lucaninae	*Nigidius miwai*	NC_063663.1	Choi et al. 2022
Lucanidae	Lucaninae	*Nigidius sinicus*	NC_065111.1	Direct submission
Lucanidae	Lucaninae	*Odontolabis cuvera fallaciosa*	PQ073195.1	Direct submission
Lucanidae	Lucaninae	*Prismognathus prossi*	NC_044962.1	Cao et al. 2019
Lucanidae	Lucaninae	*Prosopocoilus astacoides*	NC_050851.1	Xu et al. 2022
Lucanidae	Lucaninae	*Prosopocoilus astacoides blanchardi*	KF364622.1	Kim et al. 2015
Lucanidae	Lucaninae	*Prosopocoilus astacoides castaneus*	ON401054.1	Xu et al. 2022
Lucanidae	Lucaninae	*Prosopocoilus bulbosus mandibularis*	MK134566.1	Xu et al. 2022
Lucanidae	Lucaninae	*Prosopocoilus confucius*	NC_036038.1	Lin et al. 2017

Lucanidae	Lucaninae	*Prosopocoilus gracilis*	NC_027580.1	Wu et al. 2016
Lucanidae	Lucaninae	*Prosopocoilus laterotarsus*	NC_065362.1	Xu et al. 2022
Lucanidae	Lucaninae	*Prosopocoilus laterotarsus maedaorum*	MK134565.1	Xu et al. 2022
Lucanidae	Lucaninae	*Pseudorhaetus sinicus*	NC_069553.1	Zhao et al. 2021
Lucanidae	Lucaninae	*Rhaetus westwoodii*	MG159815.1	Jing et al. 2018
Lucanidae	Lucaninae	*Serrognathus castanicolor*	NC_082961.1	Jafir et al. 2024
Lucanidae	Lucaninae	*Serrognathus platymelus*	NC_044096.1	Direct submission
Lucanidae	Lucaninae	*Serrognathus titanus*	MT548769.1	Direct submission
Lucanidae	Lucaninae	*Velutinodorcus velutinus*	NC_045122.1	Direct submission
Lucanidae	Syndesinae	*Ceruchus minor*	MH120283.1	Direct submission
Lucanidae	Syndesinae	*Sinodendron rugosum*	MH120284.1	Direct submission
Lucanidae	Syndesinae	*Sinodendron yunnanense*	NC_036157.1	Lin et al. 2017
Passalidae	-	*Aceraius grandis*	MT548772.1	Direct submission
Passalidae	-	*Leptaulax koreanus*	NC_044848.1	Lee et al. 2019
Passalidae	-	*Ophrygonius sp.*	NC_060602.1	Direct submission
Scarabaeidae	Cetoniinae	*Campsiura mirabilis*	MT548771.1	Hu et al. 2023
Scarabaeidae	Cetoniinae	*Clinterocera nigra*	NC_062860.1	Wu et al. 2022
Scarabaeidae	Cetoniinae	*Coenochilus striatus*	NC_065313.1	Li et al. 2022
Scarabaeidae	Cetoniinae	*Dicronocephalus adamsi*	OK012569.1	Direct submission
Scarabaeidae	Cetoniinae	*Dicronorhina derbyana*	OK484300.1	Ayivi et al. 2021
Scarabaeidae	Cetoniinae	*Eudicella quadrimaculata*	OK484299.1	Ayivi et al. 2021
Scarabaeidae	Cetoniinae	*Eudicella smithii*	OK484302.1	Ayivi et al. 2021
Scarabaeidae	Cetoniinae	*Eudicella tetraspilota euthalia*	OK484301.1	Ayivi et al. 2021
Scarabaeidae	Cetoniinae	*Gametis jucunda*	NC_063846.1	Direct submission
Scarabaeidae	Cetoniinae	*Glycyphana fulvistemma*	NC_063847.1	Direct submission
Scarabaeidae	Cetoniinae	*Jumnos ruckeri*	OK484304.1	Ayivi et al. 2021
Scarabaeidae	Cetoniinae	*Mecynorhina polyphemus*	OK484305.1	Ayivi et al. 2021
Scarabaeidae	Cetoniinae	*Mecynorhina torquata ugandensis*	OK484306.1	Ayivi et al. 2021
Scarabaeidae	Cetoniinae	*Osmoderma caeleste*	KU500641.1	Kim et al. 2016
Scarabaeidae	Cetoniinae	*Osmoderma opicum*	NC_030778.1	Kim et al. 2016
Scarabaeidae	Cetoniinae	*Protaetia brevitarsis*	NC_023453.1	Kim et al. 2014

Scarabaeidae	Cetoniinae	*Protaetia speciosa jousselini*	OK484307.1	Ayivi et al. 2021
Scarabaeidae	Cetoniinae	*Trichius succinctus*	NC_063849.1	Direct submission
Scarabaeidae	Dynastinae	*Chalcosoma caucasus caucasus*	OK484308.1	Ayivi et al. 2021
Scarabaeidae	Dynastinae	*Dynastes hercules hercules*	OK484309.1	Ayivi et al. 2021
Scarabaeidae	Dynastinae	*Dynastes satanas*	OQ998898.1	He et al. 2024
Scarabaeidae	Dynastinae	*Eophileurus chinensis*	NC_059757.1	Direct submission
Scarabaeidae	Dynastinae	*Eupatorus gracilicornis*	NC_065036.1	Direct submission
Scarabaeidae	Dynastinae	*Eupatorus hardwickei*	NC_066495.1	Direct submission
Scarabaeidae	Dynastinae	*Eupatorus sukkiti*	NC_066494.1	Direct submission
Scarabaeidae	Dynastinae	*Megasoma elephas elephas*	OK484310.1	Ayivi et al. 2021
Scarabaeidae	Dynastinae	*Megasoma mars*	OK484311.1	Ayivi et al. 2021
Scarabaeidae	Dynastinae	*Oryctes nasicornis*	OK484312.1	Ayivi et al. 2021
Scarabaeidae	Dynastinae	*Oryctes rhinoceros*	NC_059756.1	Direct submission
Scarabaeidae	Dynastinae	*Trypoxylus dichotomus*	PQ067331.1	Direct submission
Scarabaeidae	Dynastinae	*Xylotrupes beckeri*	OK484314.1	Ayivi et al. 2021
Scarabaeidae	Dynastinae	*Xylotrupes beckeri intermedius*	OK484313.1	Ayivi et al. 2021
Scarabaeidae	Dynastinae	*Xylotrupes socrates tonkinensis*	OK484315.1	Ayivi et al. 2021
Scarabaeidae	Dynastinae	*Xylotrupes sumatrensis*	OK484316.1	Ayivi et al. 2021
Scarabaeidae	Euchirinae	*Cheirotonus gestroi*	NC_046890.1	Yang et al. 2020
Scarabaeidae	Euchirinae	*Cheirotonus jansoni*	NC_023246.1	Shao et al. 2014
Scarabaeidae	Euchirinae	*Euchirus longimanus*	OR253996.1	Direct submission
Scarabaeidae	Euchirinae	*Propomacrus bimucronatus*	NC_070352.1	Yi et al. 2024
Scarabaeidae	Euchirinae	*Propomacrus davidi*	NC_070351.1	Yi et al. 2024
Scarabaeidae	Melolonthinae	*Amphimallon sp.*	ON529251.1	Guo et al. 2022
Scarabaeidae	Melolonthinae	*Apogonia cf. basalis*	NC_065312.1	Li et al. 2022
Scarabaeidae	Melolonthinae	*Apogonia splendida*	NC_065311.1	Li et al. 2022
Scarabaeidae	Melolonthinae	*Cyphochilus crataceus*	OP963801.1	Direct submission
Scarabaeidae	Melolonthinae	*Holotrichia diomphalia*	MT548775.1	Direct submission
Scarabaeidae	Melolonthinae	*Holotrichia niponensis*	MZ726798.1	Direct submission
Scarabaeidae	Melolonthinae	*Holotrichia parallela*	MW874410.1	Direct submission
Scarabaeidae	Melolonthinae	*Miridiba trichophora*	NC_068084.1	Direct submission

Scarabaeidae	Melolonthinae	*Polyphylla gracilicornis*	NC_054285.1	Zhou et al. 2021
Scarabaeidae	Melolonthinae	*Polyphylla laticollis*	MT548774.1	Direct submission
Scarabaeidae	Melolonthinae	*Rhopaea magnicornis*	NC_013252.1	Cameron et al. 2009
Scarabaeidae	Melolonthinae	*Sophrops peronosporus*	NC_082142.1	Long et al. 2024
Scarabaeidae	Melolonthinae	*Sophrops subrugatus*	NC_065314.1	Li et al. 2022
Scarabaeidae	Rutelinae	*Anomala aulax*	NC_087770.1	Direct submission
Scarabaeidae	Rutelinae	*Anomala corpulenta*	NC_069575.1	Direct submission
Scarabaeidae	Rutelinae	*Anomala rufiventris*	NC_082143.1	Long et al. 2024
Scarabaeidae	Rutelinae	*Anomala russiventris*	NC_065310.1	Li et al. 2022
Scarabaeidae	Rutelinae	*Anomala sp.*	PQ139505.1	Direct submission
Scarabaeidae	Rutelinae	*Anomala vitalisi*	PQ139504.1	Direct submission
Scarabaeidae	Rutelinae	*Anomala xantholoma*	PQ139503.1	Direct submission
Scarabaeidae	Rutelinae	*Callistethus plagiicollis*	NC_082144.1	Long et al. 2024
Scarabaeidae	Rutelinae	*Popillia japonica*	NC_038115.1	Yang et al. 2018
Scarabaeidae	Rutelinae	*Popillia mutans*	NC_056126.1	Song & Zhang 2018
Scarabaeidae	Scarabaeinae	*Catharsius molossus*	MT548776.1	Direct submission
Scarabaeidae	Scarabaeinae	*Copris tripartitus*	NC_045923.1	Jeong et al. 2020
Scarabaeidae	Scarabaeinae	*Dichotomius schiffleri*	NC_039689.1	Amorim et al. 2017
Scarabaeidae	Scarabaeinae	*Onthophagus fodiens*	PQ067330.1	Direct submission
Silphidae	Silphinae	*Diamesus osculans*	NC_045874.1	Zhang et al. 2020
Staphylinidae	Apateticinae	*Apatetica glabra*	NC_079946.1	Direct submission
Trogidae	-	*Omorgus chinensis*	MK937809.1	Direct submission
Trogidae	-	*Trogidae sp.*	MK109856.1	Direct submission

Table S2. Nucleotide composition of the mitochondrial genome of *Lethrus scoparius*.

	**A (%)**	**C (%)**	**G (%)**	**T (%)**	**A+T (%)**	**G+C (%)**	**AT-skew**	**GC-skew**
Mitogenome	41.29	13.64	7.88	37.19	78.48	21.52	0.05	-0.27
atp6	35.86	15.63	9.08	39.43	75.30	24.70	-0.05	-0.27
atp8	42.31	13.46	3.85	40.38	82.69	17.31	0.02	-0.56
cox1	32.29	15.21	15.01	37.49	69.79	30.21	-0.07	-0.01
cox2	37.43	15.06	10.23	37.28	74.71	25.29	0.00	-0.19
cox3	33.12	15.10	13.45	38.32	71.45	28.55	-0.07	-0.06
cytb	33.77	15.49	10.76	39.98	73.75	26.25	-0.08	-0.18
nad1	29.61	7.68	15.02	47.70	77.30	22.70	-0.23	0.32
nad2	39.94	14.63	6.20	39.23	79.17	20.83	0.01	-0.40
nad3	31.07	14.12	9.89	44.92	75.99	24.01	-0.18	-0.18
nad4	30.37	7.63	13.24	48.77	79.13	20.87	-0.23	0.27
nad4l	28.87	6.19	14.09	50.86	79.73	20.27	-0.28	0.39
nad5	32.21	7.47	12.84	47.49	79.70	20.30	-0.19	0.26
nad6	34.52	12.30	10.12	43.06	77.58	22.42	-0.11	-0.10
16S rRNA	38.08	6.02	13.33	42.57	80.65	19.35	-0.06	0.38
12S rRNA	37.63	6.76	13.65	41.96	79.59	20.41	-0.05	0.34
tRNA-*Ala* (UGC)	43.94	9.09	7.58	39.39	83.33	16.67	0.05	-0.09
tRNA-*Cys* (GCA)	31.75	9.52	15.87	42.86	74.60	25.40	-0.15	0.25
tRNA-*Asp* (GUC)	44.78	7.46	5.97	41.79	86.57	13.43	0.03	-0.11
tRNA-*Glu* (UUC)	37.31	10.45	5.97	46.27	83.58	16.42	-0.11	-0.27
tRNA-*Phe* (GAA)	42.42	4.55	13.64	39.39	81.82	18.18	0.04	0.50
tRNA-*Gly* (UCC)	48.49	9.09	3.03	39.39	87.88	12.12	0.10	-0.50
tRNA-*His* (GUG)	43.94	3.03	10.61	42.42	86.36	13.64	0.02	0.56
tRNA-*Ile* (GAU)	41.79	7.46	11.94	38.81	80.60	19.40	0.04	0.23

tRNA-*Lys* (CUU)	38.03	15.49	14.08	32.39	70.42	29.58	0.08	-0.05
tRNA-*Leu* (UAG)	32.79	6.56	18.03	42.62	75.41	24.59	-0.13	0.47
tRNA-*Leu* (UAA)	37.50	14.06	14.06	34.38	71.88	28.13	0.04	0.00
tRNA-*Met* (CAU)	37.68	13.04	11.59	37.68	75.36	24.64	0.00	-0.06
tRNA-*Asn* (GUU)	47.69	9.23	12.31	30.77	78.46	21.54	0.22	0.14
tRNA-*Pro* (UGG)	39.06	6.25	14.06	40.63	79.69	20.31	-0.02	0.38
tRNA-*Gln* (UUG)	37.68	2.90	13.04	46.38	84.06	15.94	-0.10	0.64
tRNA-*Arg* (UCG)	39.39	13.64	9.09	37.88	77.27	22.73	0.02	-0.20
tRNA-*Ser* (UCU)	35.82	10.45	10.45	43.28	79.10	20.90	-0.09	0.00
tRNA-*Ser* (UGA)	43.94	6.06	10.61	39.39	83.33	16.67	0.05	0.27
tRNA-*Thr* (UGU)	45.31	4.69	9.38	40.63	85.94	14.06	0.05	0.33
tRNA-*Val* (UAC)	40.00	5.71	11.43	42.86	82.86	17.14	-0.03	0.33
tRNA-*Trp* (UCA)	46.27	11.94	8.96	32.84	79.10	20.90	0.17	-0.14
tRNA-*Tyr* (GUA)	35.29	10.29	13.24	41.18	76.47	23.53	-0.08	0.13
Control region (CR)	42.04	13.33	6.45	38.18	80.22	19.78	0.05	-0,35

References

Amorim IC, Melo ADS, Cruz GADS, Wallau GDL, Moura RDCD (2017) *Dichotomius* (*Luederwaldtinia*) *schiffleri* (Coleoptera: Scarabaeidae) mitochondrial genome and phylogenetic relationships within the superfamily Scarabaeoidea. Mitochondrial DNA Part B, 2(2), 887-888. https://doi.org/10.1080/23802359.2017.1407695

Ayivi SPG, Tong Y, Storey KB, Yu DN, Zhang JY (2021) The mitochondrial genomes of 18 new Pleurosticti (Coleoptera: Scarabaeidae) exhibit a novel trnQ-NCR-trnI-trnM gene rearrangement and clarify phylogenetic relationships of subfamilies within Scarabaeidae. Insects, 12(11), 1025. https://doi.org/10.3390/insects12111025

Cameron SL, Sullivan J, Song H, Miller KB, Whiting MF (2009) A mitochondrial genome phylogeny of the Neuropterida (lace-wings, alderflies and snakeflies) and their relationship to the other holometabolous insect orders. Zoologica Scripta, 38(6), 575-590. https://doi.org/10.1111/j.1463-6409.2009.00392.x

Cao Y, Liu J, Zhou S, Chen Y, Wan X (2019) Complete mitochondrial genome of *Prismognathus prossi* (Coleoptera: Lucanidae) with phylogenetic implications. Entomologica Fennica, 30(2), 90-96. https://doi.org/10.33338/ef.82927

Chen Y, Liu J, Cao Y, Zhou S, Wan X (2018) Two new complete mitochondrial genomes of *Dorcus* stag beetles (Coleoptera, Lucanidae). Genes & Genomics, 40, 873-880. https://doi.org/10.1007/s13258-018-0699-8

Chen YJ, Kim S, Wan X (2021) Mitochondrial genomes of the *Dorcus velutinus* complex (Coleoptera: Lucanidae) with the large intergenic spacer showing unique short sequence repeats and their implications for systematics. Journal of Asia-Pacific Entomology, 24(1), 493-501. https://doi.org/10.1016/j.aspen.2020.08.015

Choi JH, Kim CJ, Kim MS, Kang JY, Kim IK (2022) The mitochondrial genome of *Nigidius miwai* Nagel (Coleoptera: Lucanidae), a carnivorous lucanid from South Korea. Mitochondrial DNA Part B, 7(8), 1443-1444. https://doi.org/10.1080/23802359.2022.2105171

Choi JH, Kim MS, Kang JY, Kim IK, Kim CJ, Euo SS (2024) The mitochondrial genome of a carnivorous lucanid, *Figulus punctatus* Waterhouse, 1873 (Coleoptera: Lucanidae). Journal of Asia-Pacific Biodiversity. https://doi.org/10.1016/j.japb.2024.03.007

Guo S, Lin X, Song N (2022) Mitochondrial phylogenomics reveals deep relationships of scarab beetles (Coleoptera, Scarabaeidae). PLoS One, 17(12), e0278820. https://doi.org/10.1371/journal.pone.0278820

He X, Wei S, Li P, Li X (2024) Complete mitochondrial genome of the Satanas beetle, *Dynastes satanas* Moser, 1909 (Coleoptera: Scarabaeidae). Mitochondrial DNA Part B, 9(12), 1627-1631. https://doi.org/10.1080/23802359.2024.2432373

Hu L, Yang L, Tian T, Chen B (2023) Sequencing and analysis of the complete mitochondrial genome of *Campsiura mirabilis* (Coleoptera: Scarabaeidae). Acta Entomologica Sinica 66 (6), 825-834. https://doi.org/10.16380/j.kcxb.2023.06.011

Jafir M, Zhou L, Chen Y, Wan X (2024) The first mitogenomic phylogenetic framework of *Dorcus* sensu lato (Coleoptera: Lucanidae), with an emphasis on generic taxonomy in Eastern Asia. BMC Ecology and Evolution, 24(1), 66. https://doi.org/10.1186/s12862-024-02225-2

Jeong JS, Kim MJ, Kim I (2020) The mitochondrial genome of the dung beetle, *Copris tripartitus*, with mitogenomic comparisons within Scarabaeidae (Coleoptera). International Journal of Biological Macromolecules, 144, 874-891. https://doi.org/10.1016/j.ijbiomac.2019.09.165

Jin L, Ding Q, Zhang L, Bai M (2024) The first complete mitochondrial genome of the genus *Homoderus* and insights into phylogeny of Lucanidae (Coleoptera: Lucanidae). Mitochondrial DNA Part B, 9(8), 981-985. https://doi.org/10.1080/23802359.2024.2385610

Jing L, Zhou SJ, Chen YJ, Wan X (2018) Mitogenome of the monotypic genus *Rhaetus* (Coleoptera: Scarabaeidae: Lucanidae). Journal of Entomological Science, 53(4), 503-513. http://dx.doi.org/10.18474/JES17-122.1

Kim E, Kim P, An SL (2020) The complete mitochondrial genome of Korean indigenous stag beetle, *Dorcus koreanus*. Mitochondrial DNA Part B, 5(3), 3741-3742. https://doi.org/10.1080/23802359.2020.1835581

Kim MJ, Im HH, Lee KY, Han YS, Kim I (2014) Complete mitochondrial genome of the whiter-spotted flower chafer, *Protaetia brevitarsis* (Coleoptera: Scarabaeidae). Mitochondrial DNA, 25(3), 177–178. https://doi.org/10.3109/19401736.2013.792064

Kim MJ, Jeong SY, Jeong JC, Kim SS, Kim I (2016) Complete mitochondrial genome of the endangered flower chafer *Osmoderma opicum* (Coleoptera: Scarabaeidae). Mitochondrial DNA Part B, 1(1), 148-149. https://doi.org/10.1080/23802359.2016.1144104

Kim MJ, Kim KG, Kim SR, Kim I (2015) Complete mitochondrial genome of the two-spotted stag beetle, *Metopodontus blanchardi* (Coleoptera: Lucanidae). Mitochondrial DNA, 26(2), 307-309. https://doi.org/10.3109/19401736.2013.825788

Lee J, Park J, Xi H, Park J (2020) Comprehensive analyses of the complete mitochondrial genome of *Figulus binodulus* (Coleoptera: Lucanidae). Journal of Insect Science, 20(5), 10. https://doi.org/10.1093/jisesa/ieaa090

Lee SG, Choi KS, Kim CJ, Jeon HB, Kim IK (2019) Complete mitochondrial genome of *Leptaulax koreanus* (Coleoptera: Passalidae), a Korean endemic bess beetle. Mitochondrial DNA Part B, 4(1), 105-106. https://doi.org/10.1080/23802359.2018.1536492

Li Y, Nie RE, Lu Y, Lee S, Zhao Z, Wu L, Sun H, Bai, M (2022) Seven new mitochondrial genomes of phytophagous scarab beetles (Coleoptera: Scarabaeidae) and phylogenetic implications. Zootaxa, 5138(3), 324-338. https://doi.org/10.11646/zootaxa.5138.3.6

Lin ZQ, Song F, Li T, Wu YY, Wan X (2017) New mitogenomes of two Chinese stag beetles (Coleoptera, Lucanidae) and their implications for systematics. Journal of Insect Science, 17(2), 63. https://doi.org/10.1093/jisesa/iex041

Linard B, Arribas P, Andújar C, Crampton-Platt A, Vogler AP (2016) Lessons from genome skimming of arthropod-preserving ethanol. Molecular Ecology Resources, 16(6), 1365-1377. https://doi.org/10.1111/1755-0998.12539

Long T, Zhu W, Yang L, Long J, Chang Z, Chen X (2024) First report of the complete mitochondrial genome of 3 beetles (Coleoptera: Scarabaeidae) harming *Gastrodia elata* (Asparagales: Orchidaceae). Journal of Insect Science, 24(1), 12. https://doi.org/10.1093/jisesa/ieae009

Shao LL, Huang DY, Sun XY, Hao JS, Cheng CH, Zhang W, Yang Q (2014) Complete mitochondrial genome sequence of *Cheirotonus jansoni* (Coleoptera: Scarabaeidae). Genetics and Molecular Research, 13(1), 1047-1058. http://dx.doi.org/10.4238/2014.February.20.6

Sheffield NC, Song H, Cameron SL, Whiting MF (2009) Nonstationary evolution and compositional heterogeneity in beetle mitochondrial phylogenomics. Systematic Biology, 58(4), 381-394. https://doi.org/10.1093/sysbio/syp037

Song N, Zhang H (2018) The mitochondrial genomes of phytophagous scarab beetles and systematic implications. Journal of Insect Science, 18(6), 11. https://doi.org/10.1093/jisesa/iey076

Wang LJ, Huang JP, Shiao SF, Ko HP, Sung CH (2019) Characterisation of the complete mitochondrial genome of *Lucanus chengyuani* (Coleoptera: Lucanidae). Mitochondrial DNA Part B, 4(2), 3460-3462. https://doi.org/10.1080/23802359.2019.1674703

Wu LW, Chen MY, Li CL (2022) Phylogenetic position and morphological polymorphism of the chafer, *Clinterocera nigra* (Coleoptera: Scarabaeidae: Cetoniinae) from Taiwan. Mitochondrial DNA Part B, 7(8), 1513-1515. https://doi.org/10.1080/23802359.2022.2109438

Wu YY, Cao YY, Fang J, Wan X (2016) The first complete mitochondrial genome of stag beetle from China, *Prosopocoilus gracilis* (Coleoptera, Lucanidae). Mitochondrial DNA Part A, 27(4), 2633-2634. https://doi.org/10.3109/19401736.2015.1041129

Xu M, Zhou S, Wan X (2022) Phylogenetic implication of large intergenic spacers: insights from a Mitogenomic comparison of *Prosopocoilus* Stag Beetles (Coleoptera: Lucanidae). Animals, 12(13), 1595. https://doi.org/10.3390/ani12131595

Yang C, Zhu EJ, He QJ, Yi CH, Hu SJ, Wang XB (2020) Complete mitochondrial genome of the Endangered long-armed scarab *Cheirotonus gestroi* (Coleoptera: Euchiridae). Mitochondrial DNA Part B, 5(1), 869-870. https://doi.org/10.1080/23802359.2020.1715888

Yang W, Zhang Y, Feng S, Liu L, Li Z (2018) The first complete mitochondrial genome of the Japanese beetle *Popillia japonica* (Coleoptera: Scarabaeidae) and its phylogenetic implications for the superfamily Scarabaeoidea. International Journal of Biological Macromolecules, 118, 1406-1413. https://doi.org/10.1016/j.ijbiomac.2018.06.131

Yi C, Shu X, Wang L, Yin J, Wang Y, Wang Y, Zhang H, He Q, Zhao M (2024) The first report of complete mitogenomes of two endangered species of genus *Propomacrus* (Coleoptera: Scarabaeidae: Euchirinae) and phylogenetic implications. PLoS One, 19(9), e0310559. https://doi.org/10.1371/journal.pone.0310559

Zhai YN, Zhou SJ, Chen YJ, Wan X (2020) The mitochondrial genome of a rare Chinese stag beetle *Kirchnerius guangxii* (Coleoptera: Lucanidae). Mitochondrial DNA Part B, 5(2), 1633-1635. https://doi.org/10.1080/23802359.2020.1745708

Zhang X, Hou Q, Zhang L, Cai J, Meng F (2020) The complete mitochondrial genome of a potentially forensic related carrion beetle, *Diamesus osculans* (Vigors, 1825). Mitochondrial DNA Part B, 5(2), 1423-1424. https://doi.org/10.1080/23802359.2020.1736955

Zhao Z, Wang J, Wu L, Bai Y, Wang C, Qi G, Li C, Cao Y (2021) Characterization of the complete mitochondrial genome of *Pseudorhaetus sinicus* Boileau, 1899 (Coleoptera: Lucanidae). Mitochondrial DNA Part B, 6(12), 3398-3399. https://doi.org/10.1080/23802359.2021.1997656

Zhou Y, Yan J, Qi H (2021) Complete mitochondrial genome of *Polyphylla gracilicornis* (Coleoptera: Scarabaeoidea). Mitochondrial DNA Part B, 6(2), 435-436. https://doi.org/10.1080/23802359.2020.1870895
